# Simultaneous Formation of Polyhydroxyurethanes and Multicomponent Semi-IPN Hydrogels

**DOI:** 10.3390/polym16070880

**Published:** 2024-03-22

**Authors:** Ana I. Carbajo-Gordillo, Elena Benito, Elsa Galbis, Roberto Grosso, Nieves Iglesias, Concepción Valencia, Ricardo Lucas, M.-Gracia García-Martín, M.-Violante de-Paz

**Affiliations:** 1 Dpto. Química Orgánica y Farmacéutica, Facultad de Farmacia, Universidad de Sevilla, 41012 Sevilla, Spainrlucas1@us.es (R.L.); graciagm@us.es (M.-G.G.-M.); 2 Dpto. Ingeniería Química, Facultad de Ciencias Experimentales, Campus El Carmen, Universidad de Huelva, 21071 Huelva, Spain; 3 Pro2TecS—Chemical Process and Product Technology Research Center, Universidad de Huelva, 21071 Huelva, Spain

**Keywords:** NIPU, cyclic carbonates, PHU, functional polymers, interpenetrated networks, IPN, SIPN, porous materials, rheological properties

## Abstract

This study introduces an efficient strategy for synthesizing polyhydroxyurethane-based multicomponent hydrogels with enhanced rheological properties. In a single-step process, 3D materials composed of Polymer 1 (PHU) and Polymer 2 (PVA or gelatin) were produced. Polymer 1, a crosslinked polyhydroxyurethane (PHU), grew within a colloidal solution of Polymer 2, forming an interconnected network. The synthesis of Polymer 1 utilized a Non-Isocyanate Polyurethane (NIPU) methodology based on the aminolysis of bis(cyclic carbonate) (bisCC) monomers derived from 1-thioglycerol and 1,2-dithioglycerol (monomers A and E, respectively). This method, applied for the first time in Semi-Interpenetrating Network (SIPN) formation, demonstrated exceptional orthogonality since the functional groups in Polymer 2 do not interfere with Polymer 1 formation. Optimizing PHU formation involved a 20-trial methodology, identifying influential variables such as polymer concentration, temperature, solvent (an aprotic and a protic solvent), and the organo-catalyst used [a thiourea derivative (TU) and 1,8-diazabicyclo [5.4.0]undec-7-ene (DBU)]. The highest molecular weights were achieved under near-bulk polymerization conditions using TU-protic and DBU-aprotic as catalyst–solvent combinations. Monomer E-based PHU exhibited higher $\bar{M_{w}}$ than monomer A-based PHU (34.1 kDa and 16.4 kDa, respectively). Applying the enhanced methodology to prepare 10 multicomponent hydrogels using PVA or gelatin as the polymer scaffold revealed superior rheological properties in PVA-based hydrogels, exhibiting solid-like gel behavior. Incorporating monomer E enhanced mechanical properties and elasticity (with loss tangent values of 0.09 and 0.14). SEM images unveiled distinct microstructures, including a sponge-like pattern in certain PVA-based hydrogels when monomer A was chosen, indicating the formation of highly superporous interpenetrated materials. In summary, this innovative approach presents a versatile methodology for obtaining advanced hydrogel-based systems with potential applications in various biomedical fields.

## 1. Introduction

Multicomponent hydrogels, formed by combining two or more distinct polymers or other components, have garnered considerable attention for their unique properties and versatile applications in biomedicine [1,2,3,4]. Hydrogels provide a hydrated and mechanically supportive environment [1], making them cytocompatible and suitable for precise molecule release. Diverse chemical and physical approaches have been utilized to construct these hydrogel networks, incorporating both synthetic and biologically derived molecules to confer specific properties. These advancements have resulted in materials with heightened mechanical attributes and diverse biological functions, showcasing promise in tissue engineering, cancer treatment, and gene therapies [1,3]. Furthermore, the integration of modular and heterogeneous building blocks into well-defined hydrogel composites has given rise to artificial extracellular matrices (ECMs) that closely emulate the hybrid nature of natural ECMs [2]. Additionally, the progress in developing multicomponent hydrogels with stimuli-responsive properties may open avenues for applications in smart drug delivery systems (DDS) in which these hydrogels can respond to specific biomolecules through gel–sol transition behavior [4]. Nevertheless, the existing limitations in achieving these hydrogels via conventional one-step mixing protocols [4] underscore the imperative need for novel synthetic tools and methodologies. Although polyurethanes (PUs) are commonly used biocompatible and biodegradable materials [5,6] employed in medical devices [7] (pump, valves, stems, etc.) to smart materials useful in the preparation of responsive drug delivery systems [8,9], multicomponent systems involving them have not been prepared yet.

**Figure 1:**
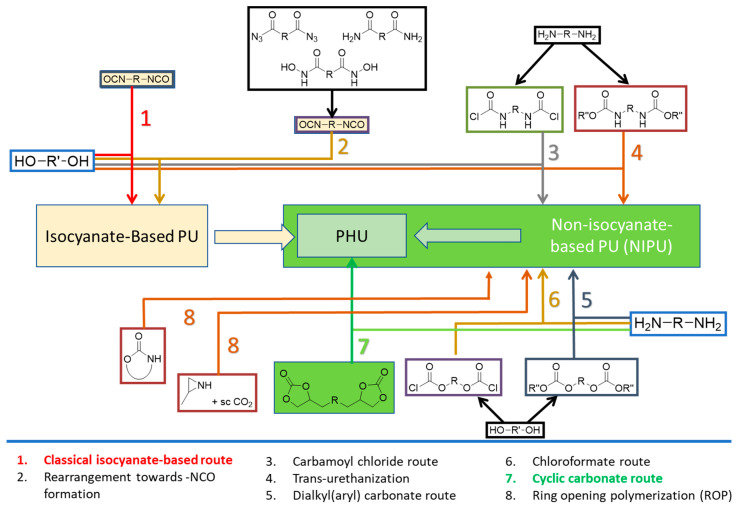
Main routes for the synthesis of polyurethanes.

PUs can be synthesized by several methods [5,6], the most relevant of which are recorded in Figure 1. Traditional PU synthesis involves reacting di(multi)isocyanates and di(multi)alcohols under moisture-free, inert atmosphere conditions using metal catalysts like stannous octoate [10]. However, these catalysts pose environmental [11] and health risks [12]. Isocyanates, used in PU synthesis, are toxic and face stringent regulations. The need for eco-friendly alternatives has led to the development of non-isocyanate polyurethanes (NIPUs) [13] with less demanding conditions, ideally at room or moderate temperatures.

NIPUs are best obtained via carbamoyl chloride, dialkyl(aryl) carbonate, chloroformate, or cyclic carbonate (CC) routes or by ring-opening polymerizations and trans-urethanizations, as shown in Figure 1 {preferred paths: routes 4 [13,14,15] and 7 [16,17,18]}.

**Scheme 1:**
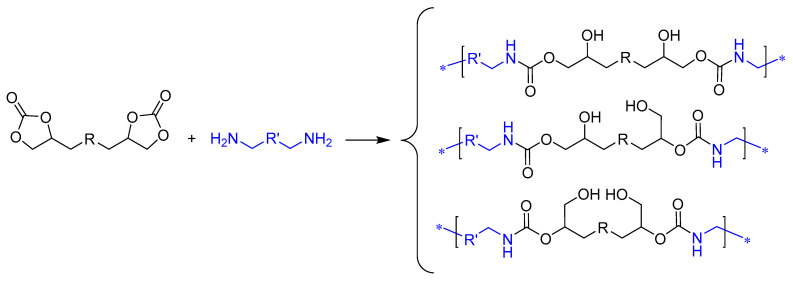
Synthesis of polyhydroxyurethanes (PHUs) from bis(cyclic carbonate)s and diamines.

The CC route (route 7, Figure 1) involves the reaction of bis(multi)CC with di(multi)amines, resulting in the opening of cyclic carbonate rings (Scheme 1). The non-regioselective ring-opening process yields polyhydroxyurethanes (PHUs) characterized by enhanced hydrophilicity compared to conventional PUs. This improved hydrophilicity offers an additional advantage for diverse biomedical applications [19]. This polymerization reaction exhibits orthogonality with a broad spectrum of biocompatible polymers and is unaltered by the presence of water or protic solvents, enabling its implementation under non-stringent mild conditions.

Multicomponent hydrogels, such as full- and semi-interpenetrating polymer networks (FIPNs and SIPNs, respectively), have emerged as innovative biomaterials for drug delivery [20] and as scaffolds for tissue engineering [21]. Typically, third-generation IPN hydrogels are constituted by two polymers whose chains are entangled with each other, giving rise to a stable and porous three-dimensional scaffold. Polymer 1 is formed within a colloidal solution of biocompatible Polymer 2 by an orthogonal polymerization method [22]. Hydrophilic polymers (natural or synthetic), such as poly(vinyl alcohol) (PVA) [23], collagen [24], and gelatin [25], are among the most suitable materials for the manufacture of such hydrogels since the presence of polar functional groups in their structure makes them soluble or swellable by water.

The primary aim of this research is to explore the potential of the cyclic carbonate-based NIPU methodology as an orthogonal tool for the efficient development of multicomponent hydrogels with enhanced rheological properties. Prior to the formation of multicomponent hydrogels, the optimal polymerization conditions, including catalyst, solvent, monomer concentration, and temperature, will be investigated for the preparation of PHUs using two new bisCC with two diamines [diethylenetriamine (DETA) and/or 1,6 hexamethylenediamine (HMDA)] as monomers. Emphasis will be placed on conducting polymerization tests at lower temperatures to produce the desired materials. The identified optimal polymerization conditions will then be applied to form multicomponent hydrogels using biocompatible hydrophilic polymers, specifically PVA and gelatin derived from porcine skin. The rheological properties of the resulting SIPNs will be thoroughly compared to investigate the influence of composition and experimental parameters.

## 2. Experimental Section

### 2.1. Materials and General Methods

All chemicals used were from Aldrich Chemical Co (Madrid, Spain). 1,2-Dithioglycerol, triethylamine (TEA), diethylenetriamine (DETA), and tris(2-aminoethyl)amine (TAEA) were stored at r.t. while 1-thioglycerol was kept in the fridge at 4 °C under argon atmosphere until needed. Gelatin porcine skin type A (PSTA) (Cat. No. G2625-100G; Lot # SLCD1818; CAS 9000-70-8; type A, derived from acid-cured tissue; ~175 bloom; 65–85% protein content by Biuret) and PVA [hydrolysis degree: 89%; weight average molecular weight ($\bar{M_{w}}$, kDa): 32.0] were also purchased from Aldrich Chemical Co (Spain) and used as received.

IR spectra were recorded on a Jasco FT/IR 4200 spectrometer (Easton, MD, USA) equipped with ATR. NMR spectra were acquired at 300 K on either a Bruker Advance AVIII-300 MHz or a Bruker AV NEO500 MHz (Frankfort, Germany) (CITIUS, Universidad de Sevilla). Chemical shifts (δ) are reported as parts per million (ppm) downfield from Me_4_Si. Mass spectra were obtained using either an IRMS Thermo Scientific Delta V Plus instrument (Waltham, MA, USA) or a Thermo Scientific Orbitrap Elite instrument (Waltham, MA, USA) (CITIUS, Universidad de Sevilla).

Gel permeation chromatography (GPC) of the samples was conducted using Waters equipment (Milford, MA, USA) provided with a refractive-index detector 2414 (thermostated at 40 °C). *N,N*-dimethylformamide (DMF) containing LiBr (5.8 mM solution) was the mobile phase. Sample preparation: DMF solution (HPLC grade) approx. 4 mg in 2.0 mL, filtration (0.22 μm), and duplicate injection of the stock solution. Samples (100 μL of 0.2% (*w*/*v*) solution) were injected and chromatographed with a flow of 1 mL·min^−1^. HR3 and HR4 Waters Styragel columns (7.8 × 300 mm) were used, linked in series, and protected with a guard column thermostated at 60 °C. Molecular mass averages and their distributions were estimated against polystyrene standards.

The thermal behavior of the polymers was examined using differential scanning calorimetry (DSC) on a TA Instruments DSC Q200 (Milford, MA, USA), which was calibrated with indium. DSC data were obtained from samples weighing 4–6 mg, with heating rates set at 10 °C per minute under a nitrogen flow. The glass transition temperatures (*T_g_*) were determined using rapidly melt-quenched polymer samples with a heating rate of 20 °C min^−1^. The testing procedure involved initially ramping up the temperature to 170 °C at a heating rate of 10 °C per minute, followed by a 2 min hold at 170 °C to eliminate the thermal history of the sample. Subsequently, they were cooled from the melt at a rate of 10 °C per minute to −30 °C and held at −30 °C for 2 min. Finally, the temperature was increased up to 170 °C at a rate of 20 °C per minute. The resulting heating curve was recorded for further analysis.

In the analysis of the multicomponent hydrogels prepared, both rheological measurements and images from field emission scanning electron microscope were conducted/recorded at the CITIUS facilities of the Universidad de Sevilla. The rheological measurements of the hydrogels were performed in a Discovery HR-3 (TA Instruments, New Castle, DE, USA) rheometer equipped with a Peltier temperature controller at 25 °C using a plate–plate geometry (diameter: 40 mm and 1 mm gap). Small-amplitude oscillatory shear (SAOS) tests from 0.12 to 62 rad/s in the linear viscoelastic regime were carried out. The linear viscoelasticity range was performed at a frequency of 1 Hz through strain amplitude sweeps. At least two replicates were performed on fresh samples. Images were recorded specifically at the Microscopy Laboratories by means of a field emission scanning electron microscope, Zeiss EVO, at an accelerating voltage of 10 kV using secondary electrons. The samples were dried using the critical point drying method to prevent the alteration of their surface topography. This protocol is widely applied for the analysis of biological tissues and was followed according to a previous study [26].

The synthesis and characterization of small molecules and monomers are described in the Supplementary Information document. All spectra of synthesized monomers and small molecules, as well as those of polymers (proton and carbon NMR, two-dimensional homo- and heteronuclear NMR spectra, ATR-FTIR spectra, and mass spectra), and the GPC chromatograms from the 30 polymerization experiments conducted under various conditions are accessible for consultation in idUS (*Depósito de Investigación de la Universidad de Sevilla*) [27].

### 2.2. Preparation of PHU by Means of Non-Isocyanate Polyurethane (NIPU) Methodology

**Scheme 2:**
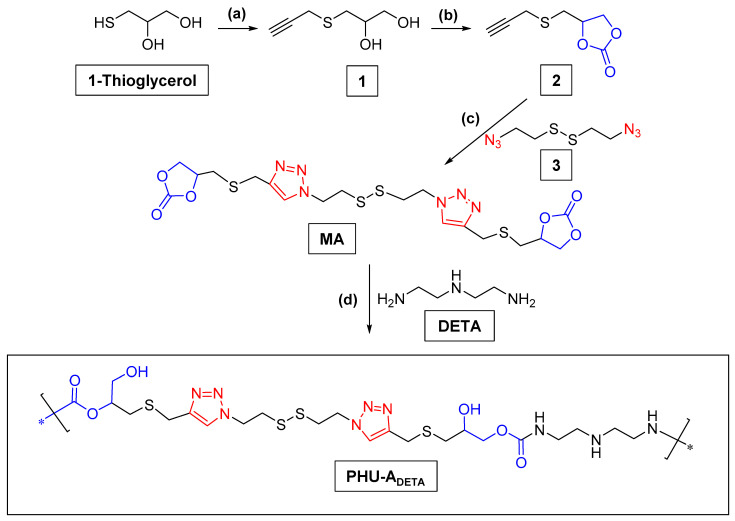
Synthetic scheme for the preparation of monomer A and PHU-A_DETA_. (a) Propargyl bromide, TEA, MeCN, 0 °C → r.t., 5 h; (b) bis(trichloromethyl) carbonate, pyridine, CH_2_Cl_2_, —55 °C → r.t., overnight; (c) diazide 3, sodium ascorbate, CuSO_4_, ^t^BuOH-H_2_O 2:1, r.t., 19 h; (d) The preparation of PHU-A_DETA_ from bis(cyclic carbonate) from MA and DETA were conducted under several reaction conditions that are summarized in Table S1.

**Scheme 3:**
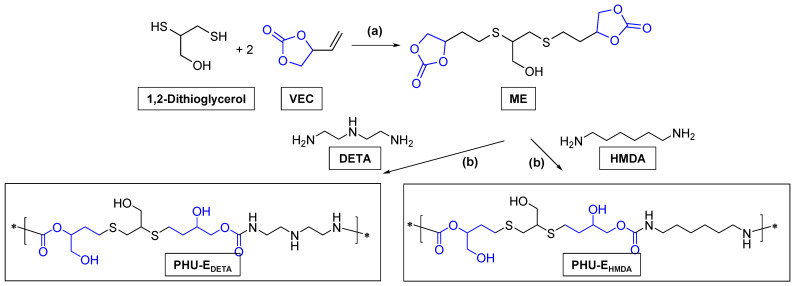
Synthetic scheme for the preparation of monomer E and PHU-E_DETA_ and PHU-E_HMDA_. (a) DMPA, CH_3_OH, UV 365 nm, r.t. (b) The polymerizations were conducted under several reaction conditions that are summarized in Table S2.

The synthesis of PHU was conducted from five-membered bis (cyclic carbonate) (bisCC) monomers (A and E) following the schemes recorded next (Scheme 2 and Scheme 3) under a batch of polymerization conditions summarized in Tables S1 and S2.

#### 2.2.1. Preparation of PHU-A_DETA_ from Monomer A and DETA

The preparation of PHU-A_DETA_ was conducted by the aminolysis of monomer A with DETA (Scheme 2) under a variety of polymerization conditions recorded (Table S1).

As an example, the general in-solution polymerization procedure for the manufacture of PHU-A_DETA_ was as follows: to a solution of monomer A (90 mg, 0.164 mmol, [monomer] = 165 mmol/L) and DETA (12 μL, 0.114 mmol) in the chosen solvent [whether DMSO or 2,2,2-trifluoroethanol (TFE)], the organo-catalyst (TU, DBU, or none) was added (8.26 μmol, 10% in mol regarding monomer A) and the reaction stirred at the chosen temperature (25 °C or 50 °C) for 24 h (Table S1, entries from 1 to 12).

Similarly, *quasi*-in-bulk polymerizations ([monomer] = 1.8 mol/L) with the same solvents, catalysts, and selected temperatures were conducted (Table S1, entries from 13 to 20). In every case, once the polymerization had taken place, the polymer was precipitated over cold *tert*-butyl methyl ether (*^t^*BME), washed with additional *^t^*BME, and dried under vacuum for 48 h (from 91% to quantitative yields). $\bar{M_{w}}$ ranged from 3.4 kDa to 16.4 kDa (Table S1, entries 13–20).

Spectroscopic data for PHU-A_DETA_: IR (ν cm^−1^) 3301 (O-H), 2933, 2863 (C-H), 1698 (C=O urethane), 1537 (N-H, N-C=O urethane). ^1^H-NMR (500 MHz, CD_3_OD) δ (ppm) 7.94 (s, 2H, H-6), 7.34–7,11 (m, 2H, N-H urethane), 4.92 (bs, 1H, H-2), 4.68 (t, 4H, *J*_7,8_ = 6.5 Hz, H-7), 4.07, 4.02 (2 bs, 4H, H-10), 3.86 (bs, 4H, H-4), 3.77–3.31 (m, 2H, H-9, H-1), 3.24 (bs, 8H, H-8, H-11), 2.85–2.50 (m, 8H, H-3, H-12). ^13^C-NMR (125 MHz, CD_3_OD) δ (ppm) 159.1 (C=O), 143.7 (C-5), 125.0 (C-6), 75.6 (C-2), 70.5 (C-9), 68.5 (C-10), 63.6 (C-1), 50.1 (C-7), 41.5 (C-8), 39.0 (C-11), 36.1 (C-12) 33.1 (C-3), 27.4 (C-4).

#### 2.2.2. Preparation of PHU-E_DETA_ and PHU-E_HMDA_

The preparation of PHU-E_DETA_ and PHU-E_HMDA_ was conducted by the aminolysis of monomer E with DETA and HMDA, respectively, under a variety of polymerization conditions (Scheme 3, Table S2). In all cases, the concentration of monomer E utilized was the highest tested for monomer A (1.8 mol/L), and the polymerization variables were temperature (25 °C or 50 °C), solvent (DMSO and EtOH as an aprotic and a protic solvent, respectively), and the organo-catalyst used (DBU or TU). The polymerization procedure was identical to that described for PHU-A_DETA_, rendering the polymeric materials with very high yields (>90% to quantitatively). $\bar{M_{w}}$ for PHU-E_DETA_ ranged from 10.3 kDa to 29.6 kDa (Table S2, entries 1–5) and from 13.6 kDa to 34.0 kDa for PHU-E_HMDA_ (Table S2, entries 6–10).

Spectroscopic data for PHU-E_DETA_: IR (ν cm^−1^) 3299 (O-H, N-H), 2930, 2857 (C-H), 1689 (C=O urethane), 1539 (N-H, N-C=O urethane). ^1^H-NMR (500 MHz, DMSO-*d_6_*) δ (ppm) 7.07, 7.03 (2 bs, 2H, N-H), 4.99–4.81 (m, 3H, OH), 4.70 (bs, 1H, H-2), 3.99–3.83 (m, 2H, H-9), 3.76 (bs, 1H, H-8), 3.71–3.64 (m, 2H, H-7), 3.62–3.50 (m, H-1), 3.14–3.04 (m, 4H, H-10), 2.94–2.81 (m, 1H, H-5), 2.80–2.68 (m, 2H, H-6), 2.67–2.58 (m, 8H, H-4, H-4′, H-11), 2.01–1.54 (m, 4H, H-3, H-3′). ^13^C-NMR (125 MHz, DMSO-*d_6_*) δ (ppm) 156.8 (C=O), 73.7 (C-2), 68.2 (C-9), 67.7 (C-8), 63.2 (C-7), 62.7 (C-1), 48.9 (C-11), 48.1 (C-5), 40.9 (C-10), 34.4, 34.2, 31.9, 31.5 (C-3, C-3′, C-6), 28.6, 27.1, 26.9 (C-4, C-4′).

Spectroscopic data for PHU-E_HMDA_: IR (ν cm^−1^) 3301 (O-H, N-H), 2929, 2860 (C-H), 1695 (C=O urethane), 1538 (N-H, N-C=O urethane). ^1^H-NMR (500 MHz, DMSO-*d_6_*) δ (ppm) 7.11, 7.06 (2 bs, 2H, N-H), 4.97–4.79 (m, 2H, OH), 4.68 (bs, 1H, H-2), 3.97–3.81 (m, 2H, H-9), 3.73 (bs, 1H, H-8), 3.70–3.61 (m, 2H, H-7), 3.60–3.51 (m, 2H, H-1), 3.07–2.96 (m, 4H, H-10), 2.95–2.80 (m, 1H, H-5), 2.79–2.66 (m, 2H, H-6), 2.46–2.58 (m, 4H, H-4, H-4′), 1.95–1.52 (m, 4H, H-3, H-3′), 1.43 (bs, 4H, H-11), 1.29 (bs, 4H, H-12). ^13^C-NMR (125 MHz, DMSO-*d_6_*) δ (ppm) 156.7 (C=O), 73.6 (C-2), 68.1 (C-9), 67.7 (C-8), 63.2 (C-7), 62.7 (C-1), 48.2 (C-5), 40.8 (C-10), 34.5, 31.9 (C-3, C-3′), 34.3 (C-6), 29,7 (C-11), 28.5, 26.7 (C-4, C-4′), 26.3 (C-12).

### 2.3. One-Step Procedure for the Manufacture of SIPN-Based Hydrogels. Characterization of the New Multicomponent Hydrogels

All multicomponent hydrogels were prepared via a single-step procedure: Polymer 1 (crosslinked PHU) was in situ synthesized within a colloidal solution of either PVA or gelatin (Polymer 2), leading to SIPNs.

In a typical procedure (preparation of IPN 4, Table 1), monomer E (700 mg, 1.98 mmol), DETA (192 μL, 1.78 mmol), the catalyst DBU (29 μL, 0.198 mmol), and the appropriate amount of the covalent crosslinker tris(2-aminoethyl)amine [TAEA, 19 μL, 0.13 mmol (degree of crosslinking aimed: 10%] were added to a colloidal solution of gelatin (900 mg) in 1:1 DMSO-H_2_O (7 mL), and the mixture was gently stirred. An additional volume of 1:1 DMSO-H_2_O solution was added (enough to a final volume equal to 9 mL). The system was gently stirred at 25 °C for 8 h and later heated up to 50 °C, and agitation was maintained for an additional 16 h. The Polymer 1/Polymer 2 ratio was 1:1 in weight.

All the SIPN systems were prepared in a manner analogous to the aforementioned process, following the reaction conditions outlined in Table 1.

The rheological properties of the hydrogels were studied and compared with the colloidal solutions of Polymer 2 on its own (blanks: entries 1–4, Table 1). In these tests, the elastic and viscous moduli ($G^{\prime }$ and $G^{\prime \prime }$, respectively) were obtained, together with the loss tangent [tan δ=G″G′ ]. Furthermore, the values for the plateau modulus ($G_{N}^{0}$) and tan (δ) at 1 rad/s were selected and tabulated to improve the comparison of the different systems (Table 1). The plateau modulus, $G_{N}^{0}$, was estimated as the $G^{\prime }$ value at a frequency for which the loss tangent is minimum [28]. It may be considered as a measure of the aggregation of the dispersed structural units or the density of physical entanglements, and, consequently, it is related to the strength of the microstructural network [29].

The microstructure of selected multicomponent hydrogels was investigated through images produced by a field emission scanning electron microscope on samples dried by the critical point drying method to avoid alteration of their surface topography.

## 3. Results and Discussion

In the present work, 10 multicomponent hydrogels with improved rheological properties have been prepared by means of a one-step process in which a crosslinked functional PHU (Polymer 1) grows within the colloidal solution of a selected biocompatible polymer (Polymer 2: gelatin type A or PVA, Table 1). Polymer 1 was formed by the aminolysis reaction of firstly synthesized 5CC (monomer A and/or monomer E) with DETA or HMDA, using TAEA as a crosslinker (PHU-A_DETA_, PHU-E_DETA,_ and PHU-E_HMDA_, Scheme 2 and Scheme 3). Optimization of such PHU production was carried out to discover relevant findings on polymerization conditions that can help in the further development of the multicomponent hydrogels targeted.

### 3.1. Synthesis of Monomer A and Preliminary Studies on PHU-A_DETA_ Formation

Monomer A, selected for its disulfide bonds and interest in developing new materials for biomedical applications [8,9], was synthesized through a three-step synthetic process from 1-thioglycerol, as outlined in Scheme 2. The nucleophilic thiol group in the starting material facilitated functionalization with a propargyl moiety (compound **1**) via an S_N_2 reaction. The formation of five- and six-membered cyclic carbonates from 1,2- and 1,-3-diols, respectively, can be carried out successfully by reaction with electrophilic carbonates [30]. In our study, the synthesis of compound **2** was successfully achieved by reacting diol 1 with bis(trichloromethyl) carbonate, resulting in the formation of propargyl CC 2 with excellent yields (96%). The versatile nature of the building block propargyl cyclic carbonate 2, obtained with an overall yield of 86.4% from 1-thioglycerol, expands the design possibilities for new CC monomers and crosslinkers. This facilitates the implementation of click azide-alkyne cycloaddition reactions involving compound **2** and diazides or multiazides, whether commercially available or synthesized de novo, to produce a diverse range of compounds suitable for PHU preparation.

In this investigation, the chosen diazide (compound **3**) features a disulfide linkage, making it responsive to the natural tripeptide glutathione [9]. The synthesis of monomer A was completed by utilizing click azide-alkyne chemistry in the final step, resulting in moderate yields.

To elucidate the impact of polymerization conditions—specifically temperature, solvent, monomer concentration, and catalyst—on PHU formation, we conducted a comparative study on the synthesis of PHU-A_DETA_. This involved the aminolysis of monomer A with DETA, as outlined in Scheme 2, under various experimental conditions (Table S1). The objective was to explore lower temperatures and eliminate the need for metal catalysts in the production of these novel materials.

**Figure 2:**
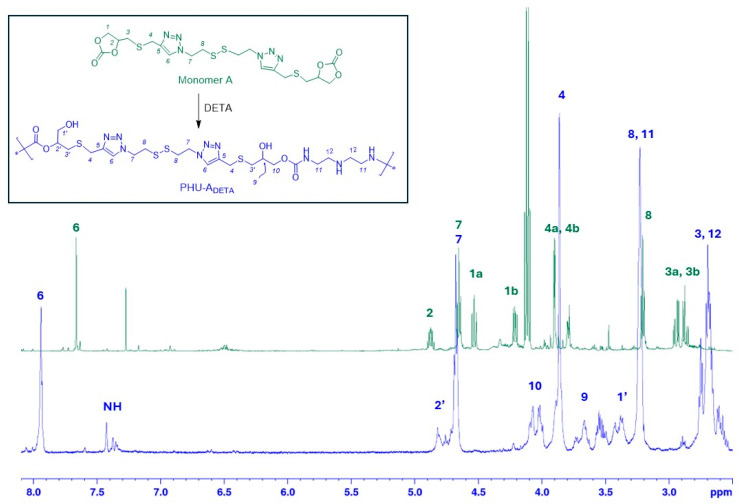
^1^H NMR of monomer A (500 MHz, CDCl_3_) and the PHU formed by reaction with DETA [PHU-A_DETA_, 500 MHz, CD_3_OD, (entry 15, Table S1)].

PHU-A_DETA_ was obtained through organo-catalyzed experiments and characterized by NMR and FTIR spectroscopies. Figure 2 presents the ^1^H NMR spectra of monomer A and of the synthesized PHU, with their structure conclusively confirmed through mono- and bi-dimensional NMR experiments (^1^H NMR, ^13^C NMR, COSY, and HSQC experiments). Detailed peak assignments are documented in the Experimental Section. Notably, the spectrum of PHU reveals not only the disappearance of peaks attributable to the CH and CH_2_ protons from the cyclic carbonate groups (at δ 5.00 ppm) but also the presence of urethane bonds, formed by the reaction between the cyclic carbonate and amine groups from DETA (the carbon of the urethane bonds appears at δ 159.1 ppm in the ^13^C NMR spectrum). Additionally, peaks corresponding to protons from the DETA monomer in the polymer backbone can be identified at δ 3.24 and 2.85–2.50 ppm.

**Figure 3:**
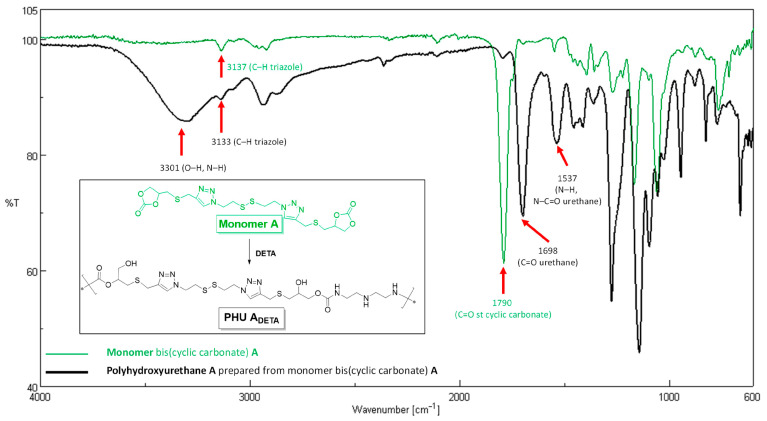
FT-IR spectra of monomer A (green) and PHU-A_DETA_ (black) (Table S1, entry 13).

To verify the formation of PHU-A_DETA_, attenuated total reflection (ATR)–Fourier Transform (FT) IR spectroscopy was employed, providing complementary evidence to NMR experiments. The ATR-FTIR spectra of monomer A and PHU-A_DETA_ are recorded in Figure 3.

The polymer spectrum confirms the opening of the cyclic carbonate ring through several indicators: Firstly, the broad band centered at ν_max_ 3301 cm^−1^, primarily attributed to the formation of primary and secondary hydroxyl groups, overlaps with the stretch bands corresponding to N-H bonds from DETA secondary amines and the newly generated urethane groups. Secondly, the presence of urethane linkages is substantiated by characteristic bands at ν_max_ 1698 cm^−1^ (associated with the stretching vibration of the C=O) and 1537 cm^−1^ [related to the stretching vibration of the N-C(=O) bond and the N-H flexion band]. Thirdly, the near disappearance of the strong band at ν_max_ 1790 cm^−1^, indicative of the vibration of C=O bonds from the cyclic carbonate monomer, conclusively validates the procedure. Notably, the band corresponding to the stretching vibration of C-H bonds from the triazole rings (≈3135 cm^−1^) is evident in both spectra.

The formation of urea by-products has been documented when methanol serves as the solvent, as evidenced by the stretching of the urea C=O group in the range of ν_max_ 1665–1630 cm^−1^ [18]. The authors attributed this phenomenon to the protic nature of the solvent. Markedly, no such occurrence was observed in any of the tested polymerization conditions, whether employing the protic solvent TFE or the aprotic solvent DMSO (refer to Figure 3, PHU-A_DETA_-13; polymerization conducted in TFE, Table S1). In summary, FTIR-based findings align with conclusions drawn from NMR spectra, confirming the efficient production of linear PHU-A_DETA_ under the specified conditions.

### 3.2. Optimization of Polymerization Conditions for PHU-A_DETA_ Formation

The formation of CC-based PHU often results in low-molecular-weight polymers due to the limited reactivity of CC with amino groups during PHU formation [19]. To improve CC aminolysis, organo-catalysts such as *N*’-[3,5-bis(trifluoromethyl)phenyl]-*N*-cyclohexylthiourea (TU), 1,5,7-triazabicyclo [4.4.0]dec-5-ene (TBD), and 1,8-diazabicyclo(5.4.0)undec-7-ene (DBU) have been employed, with TU [31] and DBU [22] exhibiting optimal results. Consequently, DBU and TU were chosen as catalysts based on their favorable performance in this polymerization reaction. Alternatively, the polymerization process typically occurs in bulk or in the presence of aprotic polar solvents such as dimethylsulfoxide (DMSO) [32], *N,N*-dimethylformamide (DMF) [17], or *N,N*′-dimethylacetamide (DMAc) [33]. DMSO has demonstrated excellence as a solvent for NIPU formation [32,34]; therefore, its inclusion in the experimental batches is warranted. Moreover, considering that strong hydrogen bonds between PHU chains are known to impede an increase in polymer molar mass [35], we advocate for the utilization of a protic solvent in this study to potentially improve PHU formation by interfering with hydrogen bonding among polymer chains. While there are limited precedents for the use of volatile protic solvents in this type of polymerization [18], exploring their potential as an alternative is justified. TFE was chosen as the protic solvent for several trials due to its high solubility of monomer A and DETA, which may positively correlate with higher PHU molecular weights [19]. While high temperatures are often employed [16], we aimed for milder conditions and ensured that polymerization temperatures did not exceed 50 °C. Interesting precedents in polymerization procedures performed at room temperature have been published [36,37].

To enhance polymerization efficiency while maintaining mild reaction conditions, we systematically assessed 20 different conditions (Table S1). Samples were designated based on their composition as PHU-A_DETA_ (monomer A and monomer DETA), with a numerical identifier distinguishing between tested conditions (from PHU-A_DETA_-1 to PHU-A_DETA_-20). The parameters explored during the polymerization encompassed the following: (a) monomer concentration, spanning from in-solution polymerizations (entries 1–12, Table S1) to near-bulk polymerizations (entries 13–20, Table S1); (b) temperature, offering choices of 25 °C or 50 °C; (c) solvent selection, featuring a protic polar solvent (TFE) or an aprotic polar solvent (DMSO); and (d) incorporation of an organo-catalyst, such as TU, DBU, or no catalyst. These variations were systematically investigated to pinpoint the optimal set of conditions for the polymerization process.

The principal aim was to evaluate the viability of the polymerization process without the involvement of a catalyst. FTIR-based kinetic studies demonstrated the non-occurrence of aminolysis of CC at room temperature, as indicated by the absence of the urethane bond-associated band at ν_max_ 1700 cm^−1^ (entries 1 and 3, Table S1). In contrast, at 50 °C (entries 2 and 4, Table S1), the consumption of CC functional groups and the formation of carbamate bonds were observed even in the absence of a catalyst, albeit resulting in low molecular weights. This observation is consistent with prior research indicating that aminolysis of CC occurs at higher temperatures, either in bulk or in solution, even when no catalyst is employed [17,18,38]. However, achieving higher molecular weights under mild conditions necessitates the inclusion of an organo-catalyst in the formulation.

Moderate molecular weights for PHU synthesis were attainable at 25 °C through quasi-bulk or in-solution polymerizations, contingent upon the inclusion of an organo-catalyst [$\bar{M_{w}}$ of 16.4 kDa and 14.3 kDa for quasi-bulk (Table S1, entry 13) and in-solution (Table S1, entry 11), respectively]. Notably, 2 out of the 20 trials conducted yielding the highest molecular weights were carried out under near-bulk polymerization conditions [entries 13 and 20, Table S1; $\bar{M_{w}}$ of 16.4 kDa and 14.7 kDa]. Under such conditions, the optimal combinations were TU-TFE and DBU-DMSO, respectively. In the case of in-solution trials, DBU emerged as the most advantageous catalyst for any solvent–catalyst combination tested.

### 3.3. Synthesis of bis(Cyclic Carbonate) Monomer E and PHU-E_DETA_ and PHU-E_HMDA_

The more polar and flexible monomer E was prepared through a click thiol-ene reaction (Scheme 3) in high yields (89%), employing a procedure previously established for other thiol-ene combinations [20]. For the formation of monomer E-based PHU, two amine-based monomers, DETA and HMDA, were employed. Similar to A_DETA_ polymers, samples were designated based on their composition (PHU-E_DETA_ and PHU-E_HMDA_, respectively) with a numerical identifier distinguishing between tested conditions (PHU-E_DETA_-1 to PHU-E_DETA_-5; PHU-E_HMDA_-1 to PHU-E_HMDA_-5).

Building upon the prior findings in monomer A-based PHU synthesis, monomer E polymerization was conducted under quasi-in-bulk conditions, chosen over in-solution conditions, resulting in elevated molecular weights compared to those obtained for monomer A-based PHU [$\bar{M_{w}}$ of 34.1 kDa and 29.6 kDa for PHU-E_HMDA_ (Table S2, entry 7) and PHU-E_DETA_ (Table S2, entry 4), respectively]. This outcome can be ascribed not only to monomer E’s improved solubility in various solvents but also to its flexibility, characteristics that can potentially enhance the interaction and subsequent reaction between the CC functional groups and the primary amino groups located at the ends of the growing polymer chains. Similar to the trials conducted with monomer A, the most favorable co-catalyst–solvent combinations were TU-protic (Table S2, entry 4) and DBU-aprotic (Table S2, entry 7). However, the highest $\bar{M_{w}}$ values were achieved when the reaction mixture was maintained at 50 °C, although satisfactory results were also obtained at room temperature (Table S2, entry 6, $\bar{M_{w}}$ 20.3 kDa).

The polymers’ thermal behavior was assessed through differential scanning calorimetry (DSC). All examined polymers were found to be amorphous (Table 2). Typically, aliphatic PHUs synthesized by this NIPU methodology (aminolysis of CC-based monomers) exhibit a *T_g_* below zero, attributed to their relatively low molecular weight [15] and the low rigidity introduced by a hydrocarbon chain (length of 4, 6, 8 carbon atoms) between the urethane groups (*T_g_* from −18 °C to −26 °C) [14]. However, the *T_g_* values observed in this study for the PHU synthesized were relatively high compared to reported values for similar PHU, likely due to the comparatively higher $\bar{M_{w}}$ of the obtained materials. Specifically, the *T_g_* of PHU-A_DETA_-13 (Table 2) was higher than its counterpart (PHU-E_DETA_-4, Table 2), both remaining below zero degrees Celsius. This distinction may be attributed to the presence of triazole groups, contributing to the stiffness of the final materials. Notably, the *T_g_* of PHU-E_HMDA_-2 was significantly above zero degrees Celsius (*T_g_* 14.2 °C).

### 3.4. One-Step Procedure for the Manufacture of PHU-Based Multicomponent Hydrogels

**Figure 4:**
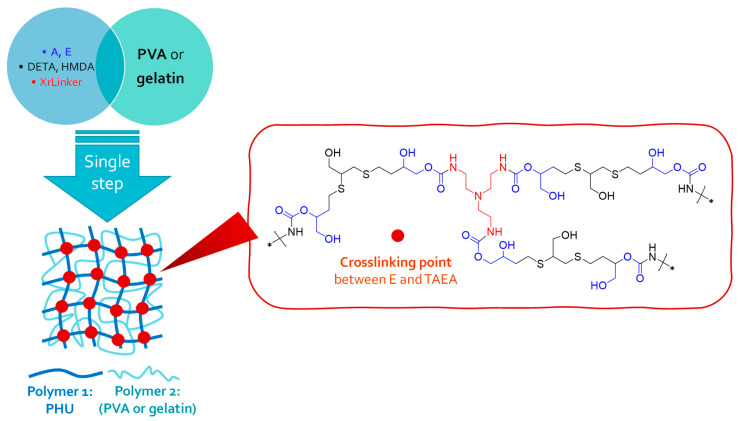
A unified approach for single-step multicomponent hydrogel synthesis.

To assess the efficacy of the described NIPU methodology for producing hybrid materials, we fabricated 10 multicomponent hydrogels based on interpenetrated networks (SIPNs) formation [Figure 4 and Table 1 (entries 5–14; Table 1 displayed in Experimental Section)]. Each procedure involved the in situ generation of a crosslinked PHU within a colloidal solution of a biocompatible polymer (either PVA or gelatin type A) to reinforce its rheological properties (Figure 4). Gelatin type A is a polydisperse mixture of low-molecular-weight polypeptides with a typical amino acid composition, including glycine (26–34%), proline (10–18%), and hydroxyproline (7–15%), collectively representing around 50% of the total amino acid content [39]. Importantly, amino acids with reactive side amino groups, which could potentially disrupt PHU formation, are negligible in gelatin A [39]. This finding implies that there are anticipated to be no substantial interferences in PHU formation, nor with PVA in PVA-based SIPNs. In general, the molecular weight of a polymer, indicative of polymer chain entanglement in solution, significantly influences the rheological properties of the solution. If there are adequate intermolecular interactions that can substitute for interchain connectivity through chain entanglements, a high molecular weight requirement may not be crucial in this scenario [40]. When considering SIPNs, their three-dimensional structure relies on the entanglement of the two polymers’ chains, leading to a collective behavior of the system. While the molecular weights of PVA and gelatin are not notably high, other critical factors contribute to the formation of intertwined materials, including the high concentration of the colloidal formulation and the development of a crosslinked PHU network within the PVA or gelatin colloidal solutions. This leads to immediate entanglement between Polymers 1 and 2.

To prepare crosslinked Polymer 1 (PHU) in PVA or gelatin solutions, the two bisCC monomers (A or E) and two diamines (DETA or HMDA) were employed, along with the crosslinking agent tris(2-aminoethyl)amine (TAEA). As monomers A and E are not soluble in water, a miscible cosolvent was employed during hydrogel formation [DMSO-H_2_O (1:1) or EtOH-H_2_O (1:1)].

**Figure 5:**
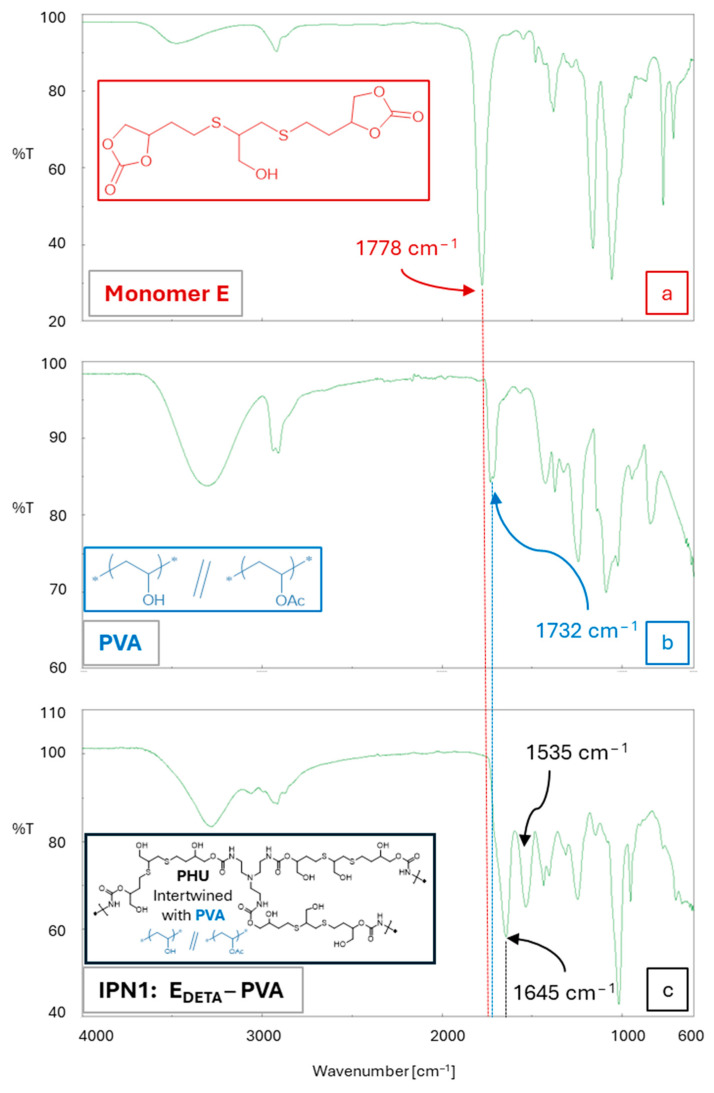
ATR-FTIR spectra of (**a**) monomer E; (**b**) PVA used in fabrication of PVA-based SIPNs; (**c**) multicomponent hydrogel IPN6 [PHU-E_DETA_/PVA].

The manufacture of the multicomponent hydrogels in a single step was confirmed through FTIR spectroscopy, as depicted in Figure 5. The presence of Polymer 1 (PHU) in the colloidal solution of Polymer 2 (PVA or gelatin) was clearly observed. Interestingly, the absence of the band at ν_max_ 1778 cm^−1^, which corresponds to the stretching band of C=O groups from CC (monomer E, Figure 5a), in the built SIPN indicates the aminolysis of these functional groups during the PHU formation process (Figure 5c). Furthermore, the IPN1 [PHU-E_DETA_/gelatin] FTIR spectrum displayed two distinct bands at ν_max_ 1645 cm^−1^ and 1535 cm^−1^ (Figure 5c). They can be attributed to the production of a crosslinked PHU network intertwined with PVA chains. Specifically, the band at 1645 cm^−1^ appears to be broadened due to its overlap with the band associated with C=O from the acetate groups present in PVA (ν_max_ 1732 cm^−1^, Figure 5b,c). These findings provide compelling evidence of the successful fabrication of SIPNs. The distinctive spectral features observed in the FTIR analysis confirm the generation of new chemical bonds and underscore the effectiveness of the SIPN formation process.

### 3.5. Characterization of Multicomponent Hydrogels

The success of this single-step procedure was further validated by studying the rheological properties of the multicomponent hydrogels and comparing them with the colloidal mixtures of Polymer 2 (Table 1). In these tests, the elastic and viscous moduli ($G^{\prime }$ and $G^{\prime \prime }$, respectively) were obtained, together with the loss tangent [tan δ=G″G′ ]. Furthermore, the values for the plateau modulus ($G_{N}^{0}$), and tan (δ) at 1 rad/s were selected and tabulated to improve the comparison of the different systems (Table 1). The plateau modulus, $G_{N}^{0}$, was estimated as the $G^{\prime }$ value at a frequency for which the loss tangent is minimum [28]. It may be considered as a measure of the aggregation of the dispersed structural units or the density of physical entanglements, and, consequently, it is related to the strength of the microstructural network [29].

The rheological properties of these hydrogels, as described in detail below (Table 1), were highly dependent on factors such as the choice of monomers, the polymer scaffold, and the cosolvent employed. Interestingly, substituting DMSO with ethanol in the solvent mixture had a dual effect. Firstly, when comparing the blanks in both solvents (entries 1 and 2 for gelatin; entries 3 and 4 for PVA), it was observed that the plateau modulus increased for both gelatin and PVA SIPNs, with a more significant impact on the former. However, when SIPNs were prepared in ethanol–water mixtures, the rheological properties were slightly enhanced for gelatin-based hydrogels (IPN3) but significantly deteriorated for PVA-based hydrogels (IPN6). Therefore, the DMSO–water mixture was preferentially used in the preparation of the remaining SIPNs.

**Figure 6:**
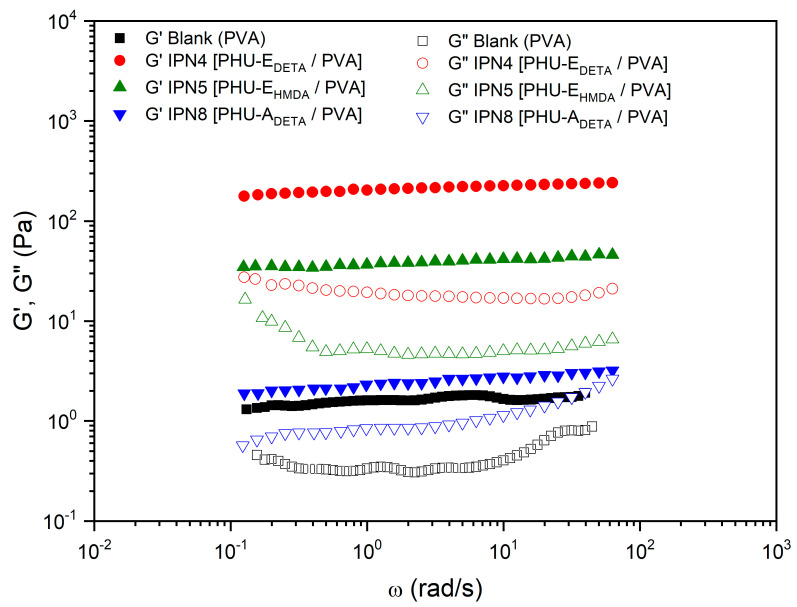
Comparative studies of the rheological properties of PVA-based SIPN and its blank. Evolution of storage modulus, $G^{\prime },$ and loss modulus, $G^{\prime \prime }$ with the frequency for SIPN hydrogels prepared by aminolysis of bisCC monomers A or E in colloidal solutions of PVA in DMSO-H_2_O 1:1. The figure legends describe the constituents of Polymer 1 (PHU) formed within the colloidal solution of Polymer 2 (PVA). DETA: diethylenetriamine; HMDA: 1,6-hexamethylenediamine; A: monomer bisCC A; E: monomer bisCC E.

Regarding the PVA-based SIPNs (in DMSO-H_2_O), all the systems, including the blank (PVA, Polymer 2, entry 3), demonstrated significantly higher values of the storage modulus ($G^{\prime }$) compared to the loss modulus ($G^{\prime \prime }$) across the entire frequency range studied (Table 1, Figure 6). This behavior is consistent with that of solid-like gels [41], where a distinct “plateau region” in the mechanical spectrum was clearly observed in all cases.

It was also verified that the diamine monomer (DETA or HMDA) did not significantly affect the evolution of the SAOS functions with frequency. However, when bisCC MA was used, a tendency to crossover between $G^{\prime }$ and $G^{\prime \prime }$ was noticed at high frequencies (Figure 6).

Moreover, monomer E exhibited remarkable potential as the preferred bisCC in producing optimized sol-like hydrogels, as evidenced by the successful formation of IPN4 [PHU-E_DETA_/PVA] and IPN5 [PHU-E_HMDA_/PVA]. Additionally, a comparative analysis of both SIPNs led to the conclusion that DETA (IPN4 [PHU-E_DETA_/PVA]) was the most suitable diamine for enhancing the SAOS functions of the blank. Consequently, DETA was employed in the formation of IPN8 [PHU-A_DETA_/PVA] in conjunction with monomer A. However, it should be noted that the utilization of monomer A in this particular case resulted in a significant reduction in the viscoelastic functions and plateau modulus.

The hydrogels IPN4 [PHU-E_DETA_/PVA] and IPN5 [PHU-E_HMDA_/PVA] demonstrated remarkably low loss tangents (0.09 and 0.14, respectively), indicating a higher degree of relative elasticity. This finding further supports the notion that these hydrogels achieved an intertwined micro-structured system, resulting in improved mechanical properties compared to the blank (colloidal solution of PVA). It was hypothesized that this enhancement occurred due not only to the crosslinking of the PHU chains through chemical crosslinking via TAEA but also the formation of stabilizing hydrogen bonds between Polymer 1 (PHU) and Polymer 2 (PVA).

The incorporation of monomer monomer A (IPN8 [PHU-A_DETA_/PVA]) resulted in slight improvements in the rheological properties of the PVA-based blank. However, these improvements were not as significant as when monomer E was chosen as the bisCC monomer in the formation of Polymer 1. This difference can be attributed to the higher stiffness of monomer A, which may have an impact on entanglements with PVA chains. Additionally, it is postulated that the lower density of free hydroxyl groups in monomer A-based PHU, compared to monomer E-PHU, substantially reduced the number of stabilizing hydrogen bonds.

**Figure 7:**
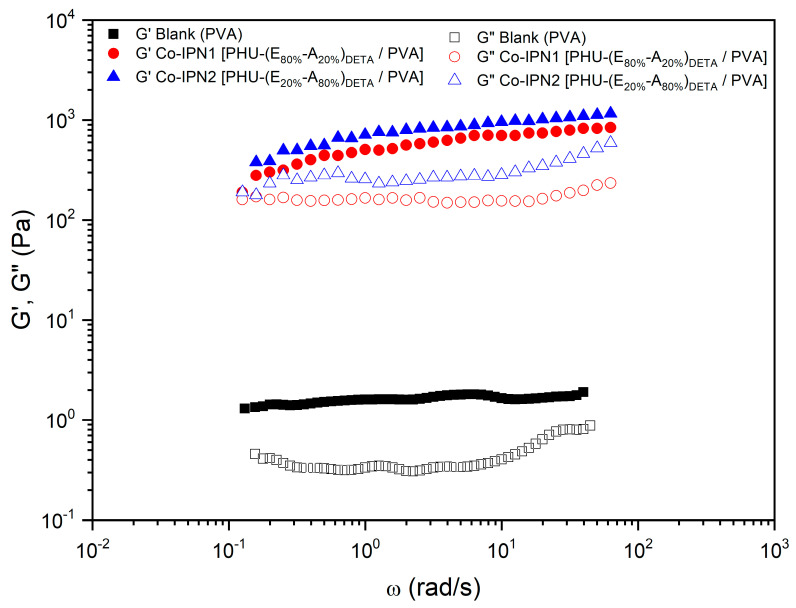
Comparative studies of the rheological properties of PVA-based co-IPN and its blank. Evolution of storage modulus, G’, and loss modulus, G”, with the frequency for co-IPN hydrogels prepared by the aminolysis of bisCC monomers A and E in colloidal solutions of PVA in DMSO-H_2_O 1:1. The figure legends describe the constituents of Polymer 1 (PHU) formed within the colloidal solution of Polymer 2 (PVA). DETA: diethylenetriamine; A: monomer bisCC A; E: monomer bisCC E.

To explore the correlation between monomer composition and rheological properties, two additional SIPNs were prepared with bisCC E/A ratios of 80:20 and 20:80 (co-IPN1 [PHU-(E_80%_A_20%_)_DETA_/PVA] and co-IPN2 [PHU-(E_20%_A_80%_)_DETA_/PVA], respectively; Figure 7). In both cases, the elastic component of the systems $G^{\prime }$ exhibited a significant increase of over two orders of magnitude compared to the blank and showed a slight frequency-dependent increase. Furthermore, $G^{\prime \prime }$ displayed a minimum and the G′ values were even higher than those observed for IPN4 [PHU-E_DETA_/PVA] (Table 1). Notably, a tendency to crossover at low frequencies was observed. Moreover, co-IPN1 [PHU-(E_80%_A_20%_)_DETA_/PVA] and co-IPN2 [PHU-(E_20%_A_80%_)_DETA_/PVA] demonstrated an increase in tan(δ) at a frequency of 1 rad/s (from 0.21 for the blank to 0.32 and 0.35 for co-IPN1 [PHU-(E_80%_A_20%_)_DETA_/PVA] and co-IPN2 [PHU-(E_20%_A_80%_)_DETA_/PVA], respectively), indicating a lower relative elasticity. These unexpected findings will be compared with ongoing studies involving various systems, further contributing to the understanding of these phenomena.

**Figure 8:**
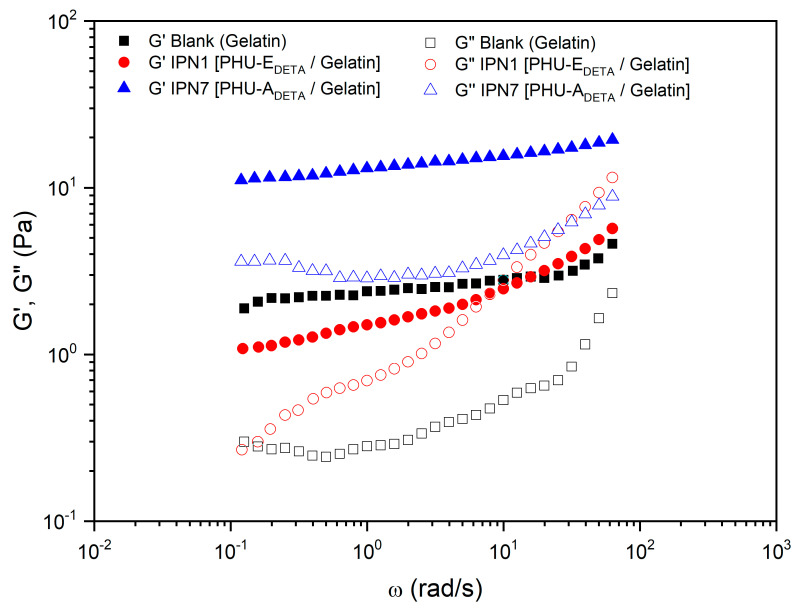
Comparative studies of the rheological properties of gelatin-based IPN and its blank. Evolution of storage modulus, G’, and loss modulus, G”, with the frequency for IPN hydrogels prepared by aminolysis of bisCC monomers A (IPN7 [PHU-A_DETA_/gelatin]) or E (IPN1 [PHU-E_DETA_/gelatin]) in colloidal solutions of gelatin in DMSO-H_2_O 1:1. The figure legends describe the constituents of Polymer 1 (PHU) formed within the colloidal solution of Polymer 2 (gelatin). DETA: diethylenetriamine; A: monomer bisCC A; E: monomer bisCC E.

Figure 8 depicts the trends observed in studies where Polymer 2 consisted of gelatin (in DMSO-H_2_O, 1:1). In these cases, IPN7 [PHU-A_DETA_/gelatin] (utilizing bisCC monomer A) exhibited higher G′ values compared to the blank (Table 1), making it the most promising in terms of rheological performance when compared to Polymer 2 alone (gelatin). This improvement can be attributed to the hydrophobic interactions between the heterocycles present in monomer A-based PHUs and the hydrophobic amino acid residues present in gelatin. In gelatin-based SIPNs, the formation of hydrogen bonds between Polymer 1 and Polymer 2 was not as significant as observed in cases where the biocompatible polymer was PVA.

On the other hand, a typical sol–gel transition response, with a crossover occurring at medium frequencies, was displayed by IPN1 [PHU-E_DETA_/gelatin]. As widely acknowledged [42], the gel strength of dispersed biopolymer systems—from dilute solutions to fully crosslinked gels—can be quantified from SAOS measurements. Firstly, the slopes of G’ and G″ versus frequency plots provide insights into the dependence of $G^{\prime }$ and G″ on frequency. Secondly, the relative values of the viscoelastic functions, particularly the loss tangent, enable the comparison of relative elasticity. Thus, regarding the loss tangent at 1 rad/s for IPN1 [PHU-E_DETA_/gelatin], a lower relative elasticity was observed, which was conclusive of a low level of crosslinking and physical entanglements.

The morphology of the IPN was studied by scanning electron microscopy (SEM). Before SEM observations, the samples underwent critical point drying (CPD) to prevent alteration of their surface topography [26]. The CPD technique is suitable for maintaining the microstructure of hydrogels due to its gentle and controlled drying process. By avoiding the abrupt phase change associated with other drying methods, such as air drying or freeze drying, CPD minimizes the formation of artifacts or structural distortions that could alter the hydrogel’s microstructure.

**Figure 9:**
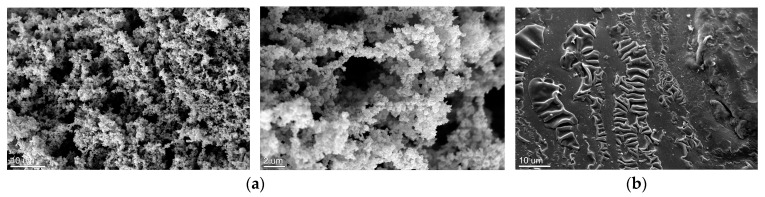
SEM images of bulk samples from monomer A-based multicomponent hydrogels. (**a**) IPN8 [PHU-A_DETA_/PVA]; (**b**): IPN7 [PHU-A_DETA_/gelatin]. Magnification 1.63 K, 5.00 K, and 1.50 K, respectively.

**Figure 10:**
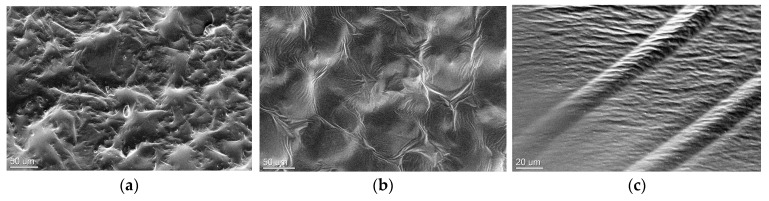
SEM images of bulk samples from ME-based SIPN (freeze-dried samples). (**a**) IPN4 [PHU-E_DETA_/PVA]; (**b**): IPN5 [PHU-E_HMDA_/PVA]; (**c**) IPN2 [PHU-E_HMDA_/gelatin]. Magnification 264, 293, and 641, respectively.

Regarding multicomponent hydrogels formed with monomer A, PVA-based IPN displayed sponge-like microstructure, as evident from the SEM images of IPN8 [PHU-A_DETA_/PVA] shown in Figure 9a. Conversely, when SEM images of gelatin-based IPN were examined, a non-porous structure was observed, as seen in the SEM images of IPN7 [PHU-A_DETA_/gelatin] shown in Figure 9b. Similar findings were observed for IPN2 [PHU-E_HMDA_/gelatin] (Figure 10c). It should be noted that these SEM photos of bulk samples may not fully capture the true internal microstructure of the hydrogel, as pointed out by other researchers in the case of gelatin-based matrices [43]. Notably, the 3D networks of ME-based multicomponent hydrogels were clearly visible when Polymer 2 was PVA, as shown in Figure 10a,b.

## 4. Conclusions

This study introduces an efficient strategy for synthesizing a single-step process to produce intertwined 3D multicomponent hydrogels (SIPNs) with enhanced rheological properties, showing promise for biomedical applications. The method demonstrates exceptional orthogonality, with the functional groups in Polymer 2 not interfering with Polymer 1 formation. Optimizing PHU formation identifies key variables, with the highest molecular weights being achieved under near-bulk polymerization conditions using TU-protic and DBU-aprotic as catalyst–solvent combinations. Monomer E-based PHU displays higher molecular weights than monomer A-based PHU (34.1 kDa and 16.4 kDa, respectively). Applying the optimized methodology to prepare 10 multicomponent hydrogels using PVA or gelatin as the polymer scaffold shows superior rheological properties in PVA-based hydrogels, exhibiting solid-like gel behavior. The incorporation of bisCC E enhances mechanical properties and elasticity (with loss tangent values of 0.09 and 0.14). SIPN hydrogels with bisCC E/A ratios of 80:20 and 20:80 demonstrate significantly increased elastic components and minimal loss components. SEM images unveil distinct microstructures, including a sponge-like pattern in certain PVA-based hydrogels when monomer A was chosen, indicating the formation of highly superporous interpenetrated materials. In summary, this innovative approach presents a versatile methodology for obtaining advanced hydrogel-based systems with potential applications in various biomedical fields.

## Figures and Tables

**Table 1:** Semi-IPN (SIPN) compositions of the hydrogels formed through the aminolysis of bisCC monomers A and E in the colloidal solution of either PVA or gelatin type A. Plateau modulus, loss tangent at 1 rad/s of the obtained PVA and gelatin-based hydrogels.

Entry	Hydrogels Prepared and Blanks	Polymer 1 (PHU) Conc.: 10% *w*/*v*		Polymer 2 (Polymer Scaffold) Conc.: 10% *w*/*v*	Solvents	Catalyst	$G_{N}^{0}{\;}^{\left(a\right)}$ (Pa)	tan (*δ*)_1_ ^(b)^
		Monomer 1 (bisCC)	Monomer 2 (Diamine)					
1	Blank: Gelatin	-	-	Gelatin	DMSO-H_2_O 1:1		2.24	0.10
2	Blank: Gelatin	-	-	Gelatin	EtOH-H_2_O 1:1		1240.46	0.12
3	Blank: PVA	-	-	PVA	DMSO-H_2_O 1:1		1.86	0.21
4	Blank: PVA	-	-	PVA	EtOH-H_2_O 1:1		11.9	0.35
5	IPN1 [PHU-E_DETA_/Gelatin]	E	DETA	Gelatin	DMSO-H_2_O 1:1	DBU	1.45	0.44
6	IPN2 [PHU-E_HMDA_/Gelatin]	E	HMDA	Gelatin	DMSO-H_2_O 1:1	DBU	4.29	0.20
7	IPN3 [PHU-E_DETA_/Gelatin]	E	DETA	Gelatin	EtOH-H_2_O 1:1	TU	5.19	0.81
8	IPN4 [PHU-E_DETA_/PVA]	E	DETA	PVA	DMSO-H_2_O 1:1	DBU	225.44	0.09
9	IPN5 [PHU-E_HMDA_/PVA]	E	HMDA	PVA	DMSO-H_2_O 1:1	DBU	40.97	0.14
10	IPN6 [PHU-E_DETA_/PVA]	E	DETA	PVA	EtOH-H_2_O 1:1	TU	8.28	0.19
11	IPN7 [PHU-A_DETA_/Gelatin]	A	DETA	Gelatin	DMSO-H_2_O 1:1	DBU	14.34	0.22
12	IPN8 [PHU-A_DETA_/PVA]	A	DETA	PVA	DMSO-H_2_O 1:1	DBU	2.39	0.38
13	co-IPN1 [PHU-(E_80%_-A_20%_)_DETA_/PVA]	E_80%_-A_20%_	DETA	PVA	DMSO-H_2_O 1:1	DBU	742.12	0.32
14	co-IPN2 [PHU-(E_20%_-A_80%_)_DETA_/PVA]	E_20%_-A_80%_	DETA	PVA	DMSO-H_2_O 1:1	DBU	958.39	0.35

(a) ($G_{N}^{0}$): Plateau modulus; (b) $\tan{\left(\delta \right)}_{1}:$ tan (δ) at 1 rad/s. DBU: 1,8-Diazabicyclo [5.4.0]undec-7-ene; DETA: diethylenetriamine; DMSO: dimethylsulfoxide; EtOH: ethanol; HMDA: 1,6-hexamethylenediamine; A: monomer bisCC A; E: monomer bisCC E; TU: *N*′-[3,5-bis(trifluoromethyl)phenyl]-*N*-cyclohexylthiourea.

**Table 2:** Comparison of $T_{g}$ values, as determined by DSC, for the three types of PHU synthesized in this study.

Sample	BisCC	Diamine	[Monomer] (mol/L)	Solvent	Temp. (°C)	Catalyst	$\bar{M_{w}}{\;}^{\left(a\right)}$ (kDa)	$T_{g}$ ^(b)^ (°C)	$T_{m}$ ^(c)^ (°C)
PHU-A_DETA_ ^(d)^	A	DETA	1.8 mol/L	TFE	25	TU	16.4	−3.0	--
PHU-E_DETA_ ^(e)^	E	DETA	1.8 mol/L	EtOH	50	TU	29.6	−10.3	--
PHU-E_HMDA_ ^(f)^	E	HMDA	1.8 mol/L	DMSO	50	DBU	34.1	14.2	--

(a) Weight average molecular weight ($\bar{M_{w}}$) calculated by gel permeation chromatography (GPC); (b) glass transition temperature ($T_{g}$) determined by differential scanning calorimetry (DSC); (c) melting temperature ($T_{m}$) determined by differential scanning calorimetry (DSC); (d) Trial 13 from Table S1; (e) Trial 4 from Table S2; (f) Trial 2 from Table S2; DBU: 1,8-Diazabicyclo [5.4.0]undec-7-ene; DETA: diethylenetriamine; DMSO: dimethylsulfoxide; EtOH: ethanol; HMDA: 1,6-hexamethylenediamine; A: monomer A; E: monomer B; TFE: 2,2,2-trifluoroethanol; TU: *N*′-[3,5-bis(trifluoromethyl)phenyl]-*N*-cyclohexylthiourea.

## Data Availability

Data will be made available on request.

## Supplementary Materials

The following supporting information can be downloaded at: https://www.mdpi.com/article/10.3390/polym16070880/s1. Table S1. Polymerization conditions tested for the preparation of PHU-A_DETA_ (aminolysis of MA with DETA) and weight average molecular weights ($\bar{M_{w}}$) achieved. Table S2. Polymerization conditions tested for the preparation of PHU-E_DETA_ and PHU-E_HMDA_ (by reaction of ME with DETA and HMDA, respectively) and weight average molecular weights ($\bar{M_{w}}$) achieved. Ref. [44] are cited in the supplementary materials.
