# Supplementary material for: Simultaneous Formation of Polyhydroxyurethanes and Multicomponent Semi-IPN Hydrogels
Source: Polymers (Basel). 2024 Mar 22;16(7):880. doi: 10.3390/polym16070880 (PMC11013152; doi:10.3390/polym16070880)
Supplement: Supplementary file 1 [file polymers-16-00880-s001.zip › polymers-2905558-supplementary.pdf]

# Supplementary information

## Simultaneous formation of polyhydroxyurethanes and multicomponent semi-IPN hydrogels

Ana I. Carbajo-Gordillo[1], Elena Benito[1]*, Elsa Galbis[1], Roberto Grosso[1], Nieves Iglesias[1], Concepción Valencia[2,3], Ricardo Lucas[1], M.-Gracia García-Martín[1], M.-Violante de-Paz[1]*

[1]Dpto. Química Orgánica y Farmacéutica, Facultad de Farmacia, Universidad de Sevilla, 41012-Sevilla, Spain.

[2]Dpto. Ingeniería Química, Facultad de Ciencias Experimentales, Campus El Carmen, Universidad de Huelva, 21071 Huelva, Spain. [3]Pro2TecS—Chemical Process and Product Technology Research Center, Universidad de Huelva, 21071 Huelva, Spain.

**Corresponding authors**: M.-Violante de-Paz (vdepaz@us.es) and Elena Benito (ebenito@us.es)

## Table of contents

**1. Synthesis and characterization of small molecules and monomers ................................2**

1.1. Synthesis of the organo-catalyst {1-[3,5-bis(trifluoromethyl)phenyl]-3-cyclohexylthiourea} (TU) ........................................................................................ 2

1.2. Synthesis of bis(cylic carbonate) monomer A (MA) from 1-thioglycerol..................... 2

1.2.1. Synthesis of 3-(prop-2-yn-1-ylthio)-propane-1,2-diol (**1**) .................................. 2

1.2.2. Synthesis of 4-[(prop-2-yn-1-ylthio)methyl]-1,3-dioxolan-2-one (**2**) ................... 3

1.2.3. Synthesis of bis(cylic carbonate) Monomer A.................................................... 4

1.3. Synthesis of bis(cylic carbonate) Monomer E ........................................................... 4

**2. Tables of Polymerization conditions tested for the formation of PHU ............................6**

**3. References ..................................................................................................7**

## 1. Synthesis and characterization of small molecules and monomers

### *1.1. Synthesis of the organo-catalyst {1-[3,5-bis(trifluoromethyl)phenyl]-3-cyclohexylthiourea} (TU)*

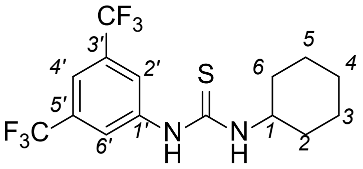


The catalyst TU was synthesized following a reported procedure [1] with slight modifications. To a solution of 3,5-di(trifluoromethyl)phenyl isothiocyanate (600 mg, 2.21 mmol) in tetrahydrofuran (THF, 10 mL), cyclohexylamine (278 $\mu$L, 2.43 mmol) was added and the solution was stirred at room temperature (r.t.) for 4 hours. The solvents were evaporated, and the resulting residue was purified by flash column chromatography on silica gel [*tert*-butyl methyl ether ($^t$BME)-hexane 1:4 → $^t$BME-hexane 1:2] to give the title compound (801 mg, 2.17 mmol, 98%) as a white solid.

IR ($\nu$ cm$^{-1}$) 3294, 3163 (N-H), 3030, 2935, 2861 (C-H), 1556 (C=S), 1526, 1468 (arom.).

$^1$H-NMR (300 MHz, $CDCl_3$) $\delta$ (ppm) 8.13 (bs, 1H, Ph-N<u>H</u>-), 7.76 (s, 2H, H-2', H-6'), 7.71 (s, 1H, H-4'), 6.07 (bs, 1H, -N<u>H</u>-cyclohexyl, 4.19 (bs, 1H, H-1), 2.07 (dd, H-2a, H-6a, $^2J_{2a,2b}$ = $^2J_{6a,6b}$ = 12.4 Hz, $J_{2a,3a}$ = $J_{6a,5a}$ 3.3 Hz), 1.70-1.10 (m, 8H, H-2b, H-3, H-4, H-5, H-6b). $^{13}$C-NMR (75 MHz, $CDCl_3$) $\delta$ (ppm) 179.3 (C=S), 138.9 (C-1'), 124.6 (C-2', C-6'), 123.9 (C-3', C-5'), 120.9 (-$CF_3$), 119.4 (C-4'), 54.0 (C-1), 32.4 (C-2, C-6), 35.3 (C-4), 24.6 (C-3, C-5).

### *1.2. Synthesis of bis(cylic carbonate) monomer A (MA) from 1-thioglycerol*

The synthesis of MA was carried out by the three-step procedure displayed in Scheme 2 starting from 1-thioglycerol.

#### *1.2.1. Synthesis of 3-(prop-2-yn-1-ylthio)-propane-1,2-diol (**1**)*

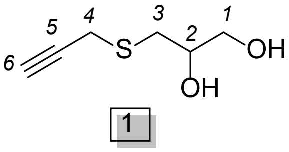


1

A solution of 1-thioglycerol (0.87 mL, 10 mmol) and TEA (1.5 mL, 11 mmol) in acetonitrile (MeCN, 5 mL) was cooled to 0 °C. An 80% w/v solution of propargyl bromide in toluene (1.2 mL, 11 mmol) was added dropwise and the solution was stirred at 25 °C for 5 h.

The reaction mixture was diluted with dichloromethane ($CH_2Cl_2$, 20 mL) and washed with double distilled water (15 mL). The aqueous phase was extracted with ethyl acetate (EtOAc, 20 mL x 3) and the combined organic phases were dried by the addition of anhydrous sodium sulfate, filtrated, and the solvents removed under reduced pressure. The residue was purified by flash column chromatography ($^t$BME-hexane 1:2) to give the title compound (**1**) as an uncolored oil (1.32 g, 90%).

IR: $\nu$ (cm$^{-1}$) 3370 (OH), 3284 (≡C-H), 2175 (C≡C).

$^{1}$H-NMR ($CDCl_3$, 500 MHz) $\delta$ (ppm) 3.93-3.89 (m, 1H, H-2), 3.79-3.75 (m, 1H, H-1a), 3.62-3.57 (m, 1H, H-1b), 3.33 (dd, 1H, H-4a, $J_{4a,6}$ = 2.7 Hz, $^{2}J_{4a,4b}$ = 17.0 Hz), 3.28 (dd, 1H, H-4b, $J_{4b,6}$ = 2.5 Hz), 2.91 (dd, 1H, H-3a, $J_{3a,2}$ = 4.0 Hz, $^{2}J_{3a,3b}$ = 13.9 Hz), 2.78 (dd, 1H, H-3b, $J_{3b,2}$ = 8.0 Hz), 2.28 (t, 1H, H-6), 2.12, 1.65 (2 bs, 2H, OH). $^{13}$C-NMR ($CDCl_3$, 125 MHz) $\delta$ (ppm) 79.7 (C-5), 71.6 (C-6), 69.9 (C-2), 65.3 (C-1), 35.4 (C-3), 19.7 (C-4).

FTMS+cESIFullms: Calculated molecular weight for $C_6H_{10}O_2NaS$ $(M + Na)^+$ 169.0294; experimental molecular weight: 169.0291.

### *1.2.2. Synthesis of 4-[(prop-2-yn-1-ylthio)methyl]-1,3-dioxolan-2-one (**2**)*

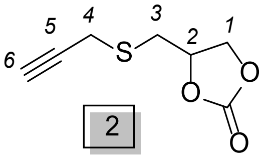


A solution of bis(trichloromethyl) carbonate (0.81 g, 2.74 mmol) in dichloromethane ($CH_2Cl_2$, 6 mL) was added dropwise to a cooled solution (-55 °C) of pyridine (2.4 mL, 32.8 mmol) and the diol **1** (0.4 g, 2.74 mmol) in $CH_2Cl_2$ (8 mL).

Once the addition was complete, the reaction was then allowed to warm to room temperature and stirred overnight. The reaction mixture was quenched with saturated aqueous ammonium chloride (20 mL) and the aqueous phase was extracted with $CH_2Cl_2$ (20 mL x 3). The combined organic extracts were washed with 1M HCl, saturated aqueous $NaHCO_3$, brine, distilled water and then dried ($Na_2SO_4$), filtered and concentrated under vacuum. The residue was purified by column chromatography ($^{t}$BME-Hex 1:1) to give the title compound (**2**) as a colored oil (0.45 g, 96%).

IR: $\nu$ (cm$^{-1}$) 3275 (≡C-H), 2919 (aliphatic C-H), 2359 (C≡C), 1788 (C=O st).

$^{1}$H-NMR ($CDCl_3$, 500 MHz) $\delta$ (ppm) 5.00-4.94 (m, 1H, H-2), 4.59 (t, 1H, H-1a, $^{2}J_{1a,1b} = J_{1a,2}$ = 8.4 Hz), 4.32 (dd, 1H, H-1b, $J_{1b,2}$ = 7.0 Hz), 3.35 (d, 2H, H-4, $J_{4,6}$ = 2.7 Hz), 3.12 (dd, 1H, H3a, $J_{3a,2}$ = 4.9 Hz, $^{2}J_{3a,3b}$ = 14.5 Hz), 2.99 (dd, 1H, H-3b, $J_{3b,2}$ = 7.0 Hz ), 2.33 (t, 1H, H-6). $^{13}$C-NMR ($CDCl_3$, 125 MHz) $\delta$ (ppm) 154.4 (C=O), 79.0 (C5), 75.3 (C-2), 72.5 (C-6), 68.6 (C-1), 33.9 (C-3), 20.2 (C-4).

FTMS+cESIFullms: Calculated molecular weight for $C_7H_8O_3NaS$ $(M + Na)^+$ 195.0086; experimental molecular weight: 195.0084.

*1.2.3. Synthesis of bis(cylic carbonate) Monomer A*

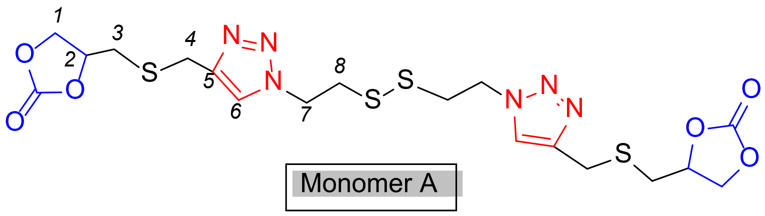


To a solution of the propargyl cyclic carbonate **2** (402 mg, 2.33 mmol) in *tert*-butanol-water 2:1 (90 mL), the freshly prepared diazide **3** ([23], 217 mg, 1.06 mmol), sodium ascorbate (46 mg, 0.233 mmol) and cupper (II) sulfate ($CuSO_4$, 8.5 mg, 0.053 mmol) were added and the solution was stirred at r.t. for 19 h. The solvent was evaporated, and the resulting residue was purified by flash column chromatography on silica gel ($CH_2Cl_2$ → $CH_2Cl_2$-MeOH 10:1, 0.5% TEA) to give monomer A (271 mg, 0.495 mmol, 46.7%) as a colorless oil. The azido-cyclic carbonate derived from the functionalization of one of the propargyl unit was also isolated (161 mg, 0.429 mmol, 40.5%).

IR (ν $cm^{-1}$) 3137 (C-H triazole), 2922 (aliphatic C-H), 1790 (C=O st cyclic carbonate).

$^1$H-NMR (500 MHz, $CDCl_3$) δ (ppm) 7.65 (s, 2H, H-6), 4.92-4.83 (m, 2H, H-2), 4.66 (t, 4H, $J_{7,8}$ = 6.5 Hz, H-7), 4.53 (t, 2H, $^2J_{1a,1b}$ = $J_{1a,2}$ = 8.5 Hz, H-1a), 4.21 (dd, 2H, $J_{1b,2}$ = 6.5 Hz, H-1b), 3.92 (d, 2H, $^2J_{4a,4b}$ = 14.5 Hz, H-4a), 3.89 (d, 2H, H-4b), 3.21 (t, 4H, H-8), 2.95 (dd, 2H, $J_{3a,2}$ = 5.0 Hz, $^2J_{3a,3b}$ = 14.0 Hz, H-3a), 2.87 (dd, 2H, $J_{3b,2}$ = 6.5 Hz, H-3b). $^{13}$C-NMR (125 MHz, $CDCl_3$) δ (ppm) 154.6 (C=O), 144.9 (C-5), 123.1 (C-6), 75.5 (C-2), 68.6 (C-1), 48.7 (C-7), 37.9 (C-8), 34.1 (C-3), 26.7 (C-4).

HRESIMS *m/z* found 571.0531, calc. for $C_{18}H_{24}N_6O_6NaS_4$ $[M+Na]^+$: 571.0532.

*1.3. Synthesis of bis(cylic carbonate) Monomer E*

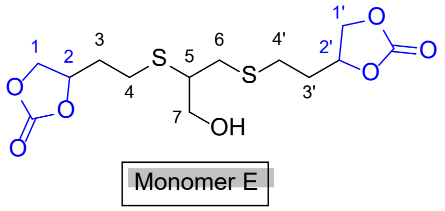


4-Vinyl-1,3-dioxolan-2-one (or vinyl-ethylcarbonate, VEC, 0.96 mL, 10 mmol) and 2,3-dimercapto-1-propanol (or 1,2-dithio-glycerol, 0.40 mL, 4 mmol) were dissolved in 5 mL of methanol.

The solution was degassed for 10 minutes. Then, 2,2-dimethoxy-2-phenylacetophenone (DMPA, 205 mg, 0.8 mmol) was added and the solution was exposed for 10 minutes at UV radiation (365 nm, 180 W) and the reaction solution was stirred overnight at r.t. The solvent was evaporated, and the crude product was purified by column chromatography on silica gel ($^t$BME → $^t$BME-MeOH 5:1) to give monomer E as a pure, slightly colored oil (1.254 g, 3.56 mmol, 89%).

IR (ν $cm^{-1}$) 3479 (OH), 2922 (aliphatic C-H), 1776 (C=O).

$^{1}$H-NMR (300 MHz, $CDCl_3$) δ (ppm) 4.99-4.85 (m, 2H, H-2, H-2'), 4.61 (t, 2H, $J_{1a,2}$ = 8.6 Hz, H-1a, H-1'a), 4.20-4.09 (m, 2H, H-1b, H-1'b), 3.85-3.70 (m, 2H, H-7), 3.50 (bs, 1H, O-H), 2.99-2.88 (m, 1H, H-5), 2.88-2.63 (m, 6H, H-4, H-4' and H-6), 2.22-2.06 (m, 2H, H-3a, H-3'a), 2.05-1.90 (m, 2H, H-3b, H-3'b). $^{13}$C-NMR (75 MHz, $CDCl_3$) δ (ppm) 154.7 (C=O), 75.4 (C-2, C-2'), 69.2 (C-1, C-1'), 63.4 (C-7), 49.0 (C-5), 34.5 (C-3, C-3'), 28.1 and 26.4 (C-4, C-4' and C-6).

FTMS+cESIFullms: Calculated molecular weight for $C_{13}H_{20}O_7NaS_2$ $(M + Na)^+$: 375.0543; experimental molecular weight: 375.0537.

## 2. Tables of Polymerization conditions tested for the formation of PHU

**Table S1**. Polymerization conditions tested for the preparation of PHU-$A_{DETA}$ (aminolysis of MA with DETA) and weight average molecular weights ($\overline{M_w}$) achieved.

| Entries | Samples PHU-$A_{DETA}$ | [Monomer] | Solvent | Temp. (°C) | Catalyst | $M_w$[(a)] (Da) |
|---|---|---|---|---|---|---|
| 1 | $A_{DETA}$-1 | 165 mmol/L | TFE | 25 | -- | -- |
| 2 | $A_{DETA}$-2 | | TFE | 50 | -- | 3,700 |
| 3 | $A_{DETA}$-3 | | DMSO | 25 | -- | -- |
| 4 | $A_{DETA}$-4 | | DMSO | 50 | -- | 3,400 |
| 5 | $A_{DETA}$-5 | | TFE | 25 | TU | 7,700 |
| 6 | $A_{DETA}$-6 | | TFE | 50 | TU | 6,800 |
| 7 | $A_{DETA}$-7 | | DMSO | 25 | TU | 6,500 |
| 8 | $A_{DETA}$-8 | | DMSO | 50 | TU | 5,400 |
| 9 | $A_{DETA}$-9 | | TFE | 25 | DBU | 13,800 |
| 10 | $A_{DETA}$-10 | | TFE | 50 | DBU | 9,600 |
| 11 | $A_{DETA}$-11 | | DMSO | 25 | DBU | 14,300 |
| 12 | $A_{DETA}$-12 | | DMSO | 50 | DBU | 8,700 |
| 13 | $A_{DETA}$-13 | 1.8 mol/L | TFE | 25 | TU | 16,400 |
| 14 | $A_{DETA}$-14 | | TFE | 50 | TU | 8,500 |
| 15 | $A_{DETA}$-15 | | DMSO | 25 | TU | 10,800 |
| 16 | $A_{DETA}$-16 | | DMSO | 50 | TU | 7,900 |
| 17 | $A_{DETA}$-17 | | TFE | 25 | DBU | 8,600 |
| 18 | $A_{DETA}$-18 | | TFE | 50 | DBU | 9,000 |
| 19 | $A_{DETA}$-19 | | DMSO | 25 | DBU | 11,200 |
| 20 | $A_{DETA}$-20 | | DMSO | 50 | DBU | 14,700 |

(a) Weight average molecular weight ($\overline{M_w}$) calculated by gel permeation chromatography.
DBU: 1,8-Diazabicyclo[5.4.0]undec-7-ene; DMSO: dimethylsulfoxide;
TFE: 2,2,2-trifluoroethanol; TU: *N'*-[3,5-bis(trifluoromethyl)phenyl]-*N*-cyclo-hexylthiourea.

**Table S2**. Polymerization conditions tested for the preparation of PHU-$E_{DETA}$ and PHU-$E_{HMDA}$ (by reaction of ME with DETA and HMDA, respectively) and weight average molecular weights ($\overline{M_w}$) achieved.

| Entries | Samples PHU-$E_{DETA}$ | Diamine | Solvent | Temp. (°C) | Catalyst | $M_w$[(a)] (Da) |
|---|---|---|---|---|---|---|
| **1** | **$E_{DETA}$-1** | DETA | DMSO | 50 | TU | 21,800 |
| **2** | **$E_{DETA}$-2** | | DMSO | 50 | DBU | 13,800 |
| **3** | **$E_{DETA}$-3** | | EtOH | 25 | TU | 14,500 |
| **4** | **$E_{DETA}$-4** | | EtOH | 50 | TU | 29,600 |
| **5** | **$E_{DETA}$-5** | | EtOH | 50 | DBU | 10,300 |
| **Entries** | **Samples PHU-$E_{HMDA}$** | **Diamine** | **Solvent** | **Temp. (°C)** | **Catalyst** | **$M_w$[(a)] (Da)** |
| **6** | **$E_{HMDA}$-1** | HMDA | DMSO | 25 | DBU | 20,300 |
| **7** | **$E_{HMDA}$-2** | | DMSO | 50 | DBU | 34,100 |
| **8** | **$E_{HMDA}$-3** | | DMSO | 50 | TU | 18,500 |
| **9** | **$E_{HMDA}$-4** | | EtOH | 50 | TU | 13,600 |
| **10** | **$E_{HMDA}$-5** | | EtOH | 50 | DBU | 13,700 |

(a) Weight average molecular weight ($\overline{M_w}$) calculated by gel permeation chromatography. DBU: 1,8-Diazabicyclo[5.4.0]undec-7-ene; DMSO: dimethylsulfoxide; EtOH: ethanol; TU: *N'*-[3,5-bis(trifluoromethyl)phenyl]-*N*-cyclohexylthiourea.

## 3. References


1. Blain, M.; Yau, H.; Jean-Gérard, L.; Auvergne, R.; Benazet, D.; Schreiner, P.R.; Caillol, S.; Andrioletti, B. Urea-and thiourea-catalyzed aminolysis of carbonates. *ChemSusChem* **2016**, *9*, 2269–2272, doi:10.1002/cssc.201600778.
2. Wang, Y.; Zhang, R.; Xu, N.; Du, F.S.; Wang, Y.L.; Tan, Y.X.; Ji, S.P.; Liang, D.H.; Li, Z.C. Reduction-degradable linear cationic polymers as gene carriers prepared by Cu(I)-catalyzed azide-alkyne cycloaddition. *Biomacromolecules* **2011**, *12*, 66–74, doi:10.1021/bm101005j.
